# Heterogeneous impact of Sighs on mortality in patients with acute hypoxemic respiratory failure: insights from the PROTECTION study

**DOI:** 10.1186/s13613-024-01385-0

**Published:** 2024-10-05

**Authors:** Emanuele Rezoagli, Carla Fornari, Roberto Fumagalli, Giacomo Grasselli, Carlo Alberto Volta, Paolo Navalesi, Rihard Knafelj, Laurent Brochard, Antonio Pesenti, Tommaso Mauri, Giuseppe Foti, for the Pleural Pressure Working Group (PLUG), Riccardo Colombo, Andrea Cortegiani, Jian-Xin Zhou, Rocco D’Andrea, Italo Calamai, Ánxela Vidal González, Oriol Roca, Domenico Luca Grieco, Tomas Jovaisa, Dimitrios Bampalis, Tobias Becher, Denise Battaglini, Huiqing Ge, Mariana Luz, Edgard Santos, Jean-Michel Constantin, Marco Ranieri, Claude Guerin, Jordi Mancebo, Paolo Pelosi

**Affiliations:** 1 School of Medicine and Surgery, University of Milano-Bicocca, 20900 Monza, Italy; 2 Department of Emergency and Intensive Care, Fondazione IRCCS San Gerardo dei Tintori, Monza, Italy; 3 Research Centre On Public Health, University of Milano-Bicocca, Monza, Italy; 4 Department of Anesthesia and Intensive Care Medicine, ASST Grande Ospedale Metropolitano Niguarda, Milan, Italy; 5 Department of Pathophysiology and Transplantation, University of Milan, Milan, Italy; 6 Department of Anesthesia, Intensive Care and Emergency, Fondazione IRCCS Ca’ Granda Ospedale Maggiore Policlinico, Milan, Italy; 7 Department of Translational Medicine, University of Ferrara, Ferrara, Italy; 8 Anesthesia and Intensive Care Unit, Emergency Department, Azienda Ospedaliera Universitaria di Ferrara, Ferrara, Italy; 9 Institute of Anesthesia and Intensive Care, Padua University Hospital, Padua, Italy; 10 Department of Medicine (DIMED), University of Padua, Padua, PD Italy; 11 Center for Internal Intensive Medicine, University Medical Center Ljubljana, Ljubljana, Slovenia; 12 Interdepartmental Division of Critical Care Medicine, University of Toronto, Toronto, Canada; 13 Keenan Research Centre for Biomedical Science, Li Ka Shing Knowledge Institute, St Michael’s Hospital, Unity Health Toronto, Toronto, Canada

**Keywords:** Acute hypoxemic respiratory failure, Pressure support ventilation, Sigh, Mortality, Extubation, Ventilatory ratio

## Abstract

**Background:**

Sigh breaths may impact outcomes in acute hypoxemic respiratory failure (AHRF) during assisted mechanical ventilation. We investigated whether sigh breaths may impact mortality in predefined subgroups of patients enrolled in the PROTECTION multicenter clinical trial according to: 1.the physiological response in oxygenation to Sigh (responders versus non-responders) and 2.the set levels of positive end-expiratory pressure (PEEP) (High vs. Low-PEEP). If mortality differed between Sigh and No Sigh, we explored physiological daily differences at 7-days.

**Results:**

Patients were randomized to pressure support ventilation (PSV) with Sigh (Sigh group) versus PSV with no sigh (No Sigh group). (1) Sighs were not associated with differences in 28-day mortality in responders to baseline sigh-test. Contrarily-in non-responders-56 patients were randomized to Sigh (55%) and 28-day mortality was lower with sighs (17%vs.36%, log-rank p = 0.031). (2) In patients with PEEP > 8cmH_2_O no difference in mortality was observed with sighs. With Low-PEEP, 54 patients were randomized to Sigh (48%). Mortality at 28-day was reduced in patients randomised to sighs (13%vs.31%, log-rank p = 0.021). These findings were robust to multivariable adjustments. Tidal volume, respiratory rate and ventilatory ratio decreased with Sigh as compared with No Sigh at 7-days. Ventilatory ratio was associated with mortality and successful extubation in both non-responders and Low-PEEP.

**Conclusions:**

Addition of Sigh to PSV could reduce mortality in AHRF non-responder to Sigh and exposed to Low-PEEP. Results in non-responders were not expected. Findings in the low PEEP group may indicate that insufficient PEEP was used or that Low PEEP may be used with Sigh. Sigh may reduce mortality by decreasing physiologic dead space and ventilation intensity and/or optimizing ventilation/perfusion mismatch.

*Clinical Trial Registration*: ClinicalTrials.gov; Identifier: NCT03201263.

**Supplementary Information:**

The online version contains supplementary material available at 10.1186/s13613-024-01385-0.

## Background

Occasional spontaneous deep breathing—known as sigh—is a physiological feature in healthy subjects during spontaneous ventilation. The first physiological characterization of sighs in healthy subjects dates back more than 100 years ago [1]. During normal breathing, sighs seem to play a key role in the prevention of atelectasis [2–4] and experimental data suggest that sighs improve the secretion of active alveolar surfactant [5].

The use of sighs during controlled mechanical ventilation was proposed to improve respiratory mechanics and gas exchange in patients undergoing surgical procedures [6] or in the presence of respiratory failure (ARDS) [7]. A renewed interest on the use of sigh during passive ventilation was recently reported in the setting of trauma patients at risk of developing ARDS for its potential benefit on outcome [8].

Sigh was also implemented during spontaneous breathing. In pressure support ventilation (PSV) – one among the most used modes of assisted mechanical ventilation [9]—Sighs improved respiratory mechanics and oxygenation, while decreasing lung heterogeneity, respiratory drive and effort [10, 11]. In 2021, the PROTECTION trial explored the feasibility of the application of sighs in PSV with acute hypoxemic respiratory failure (AHRF) or ARDS. Sigh was proved feasible and safe in this population but no differences on outcomes were reported between PSV with or without sigh breathing [12]. However, increasing awareness is emerging on the importance of phenotyping patients that may benefit the most from a therapeutic intervention based on clinical, laboratory, imaging or physiological criteria [13–15]. This may allow to reduce sample heterogeneity, leading to heterogeneity of treatment effects. Further, this may optimize the population enrichment of targeted subjects that are most likely to positively respond to a specific treatment in terms of hard outcomes.

In this secondary analysis of the PROTECTION trial we aimed at exploring the role of sigh breathing during PSV in specific predefined physiological subgroups of patients on outcomes.

These analyses may serve as preliminary, exploratory and hypotheses generating to understand whether the use of Sigh may be a ventilatory option based on the physiological response in oxygenation to Sigh and in regard to the set levels of PEEP. We based our analyses on a physiological rationale.

We started from the hypothesis that sigh breathing may be beneficial on outcome in the presence of oxygenation response during the sigh test (responders)—which was defined by SpO_2_/FiO_2_ criteria > 1%—as compared with No Sigh. Therefore, we explored differences on outcome between Sigh and No Sigh treatment (primary outcome).

Subsequently, we hypothesized that patients exposed to low levels of PEEP (PEEP ≤ 8cmH_2_O—PEEP = 8cmH_2_O defines two size balanced subgroups in the PROTECTION trial and seemed clinically reasonable [9]—Low PEEP group) may show a lower mortality rate by adding Sigh as compared with No Sigh (primary outcome).

In the presence of mortality differences between Sigh and No Sigh, we explored daily differences in physiological parameters between the 2 randomized groups, and whether physiological parameters were associated with outcomes (secondary outcomes).

## Methods

### Patients, study design and setting

These are prespecified secondary analyses of an international, multicenter, randomized clinical trial (NCT03201263) [12] aimed at exploring predefined physiological subgroups of patients potentially responsive to sighs in terms of outcomes. Further, we investigated whether differences in respiratory physiology might have a role as underpinning mechanisms of outcomes differences by using sigh.

The PROTECTION trial included 20 centers from 8 countries between December 2017 to May 2019 through a call of the Pleural Pressure Working Group (PLUG) of the European Society of Intensive Care Medicine (ESICM) who endorsed and partially funded the trial.

The PROTECTION trial included patients with acute hypoxemic respiratory failure (AHRF) (PaO_2_/FiO_2_ ≤ 300 with a PEEP of 5 cmH_2_O) who were mechanically ventilated between 24 h and 7 days and who were switched from mechanical ventilation to pressure support ventilation between 4 and 24 h. Furthermore, at the enrolment, the Richmond Agitation-Sedation Scale was −2 to 0 [16].

Further details about study design, population, exclusion criteria and methods were previously described [17].

### Sigh test, randomization, interventions and spontaneous breathing trial

All enrolled patients underwent a responsivity test to Sigh. Specifically, patients were exposed for 30 min to Sigh (i.e. 30 cmH_2_O for 3-s insufflation one each minute) starting with a FiO_2_ tailored to target a SpO_2_ between 90 and 96%. After the Sigh test, patients were defined as Sigh responders versus Sigh non-responders whether SpO_2_/FiO_2_ improved by > 1%.

After completion of the Sigh test, patients were randomized to PSV with Sigh (Sigh group) or to PSV with no sigh (No Sigh group).

PSV setting after randomization targeted a Vt 6–8 mL/kg of predicted body weight, respiratory rate (RR) 20–35 breaths/minute, while clinical PEEP and FiO_2_ were unchanged.

### In the Sigh group, Sigh was promptly added as a pressure control breath at total end-inspiratory

Pressure of 30 cmH_2_O for 3 s delivered once per minute. Ventilators were switched to biphasic synchronized positive airway pressure mode (also known as synchronized intermittent mandatory ventilation combining pressure control and PSV) with the lower pressure level set at clinical PEEP and the higher pressure level set at 30 cmH_2_O with a 3-s inspiratory time. Sigh settings were left unchanged until switch to controlled ventilation, day 28, death, or performance of a successful spontaneous breathing trial. In the No Sigh group, after randomization, PSV was set to obtain the same targets as above with clinical PEEP and the FiO_2_ selected during the prerandomization sigh test. Subsequent changes in PSV in both groups, were considered at least every 8 h to reach the randomization target of Vt and RR, while PEEP and FiO_2_ were adjusted to maintain SpO_2_ 90–96%. In both groups, switch to protective controlled ventilation was considered when in the presence of specific predefined criteria. Patients switched to controlled ventilation were reassessed at least every 8 h and switched back to the Sigh or No Sigh group as soon as predefined criteria for improvement were met [17].

A spontaneous breathing trial (SBT) was considered if SpO_2_ ≥ 90% on FiO_2_ ≤ 0.4 and PEEP ≤ 5 cmH_2_O with no agitation and unstable hemodynamics. In the sigh group, the attending physician withdrew sigh, waited 60 min, confirmed the above-mentioned criteria, and performed the SBT. If criteria were no longer met, sigh was reintroduced and this procedure was repeated after at least 8 h. The SBT lasted at least 60 min with a combination of PEEP of 0 to 5 cmH_2_O and PSV level of 0 to 5 cmH_2_O. Criteria for success vs failure of the SBT were predefined by study protocol [17]. After successful completion of the SBT, patients were promptly extubated or, in the presence of tracheostomy, mechanical ventilation was discontinued. After SBT failure, patients were switched back to the Sigh or No Sigh group, and criteria for SBT were checked again after at least 6 h. After extubation, reintubation was performed if at least one of the criteria predefined by the study protocol was present [17].

Comprehensive information on randomization, interventions and SBT was previously described [17]. The complete study protocol is included in the Supplemental material.

### Predefined physiological subgroups

Responders versus non-responders were defined based on the 30-min Sigh test based on oxygenation criteria, and—as previously explained—patients were defined as Sigh responders versus Sigh non-responders whether SpO_2_/FiO_2_ improved by > 1%.

The specific cut-off used to define High versus Low PEEP group was decided based on statistical reasons (i.e. to obtain balanced samples between the 2 predefined subgroups) and on baseline oxygenation criteria (i.e. patients with mild hypoxemia, average 200 < PaO_2_/FiO_2_ ≤ 300).

### Measurements and study outcomes

After enrolment and at randomization, data on demographics, past and recent medical history, systemic severity, lung injury risk factors, ventilation clinical settings and etiology of AHRF were collected. Furthermore, daily physiological measurements were collected during the first 7 days after randomization. Study outcomes including 28-day mortality and successful extubation with more than 48 h free from reintubation at 28-day and data on sigh feasibility were explored.

### Statistical analysis

Continuous data were described with median and quartiles (Q_1_–Q_3_). Categorical data were reported as count (proportion). Descriptive statistics were used to characterize the study population. A two-tailed p-value below 0.05 was considered statistically significant. Differences between the randomized groups (Sigh versus No Sigh groups) are reported by Mann–Whitney Wilcoxon-test and by Chi-square or Fisher’s exact test, as appropriate.

Differences in 28-day mortality and successful extubation with more than 48 h free from reintubation at 28-day were evaluated by survival curves using the Kaplan–Meier approach with log-rank p-value and competing risk non-parametric method with Fine & Gray p-value, respectively. The association of the study intervention (Sigh versus No Sigh) with 28-day mortality and successful extubation was investigated by using multivariable Cox-proportional and Fine & Gray models using mortality as a competitive event, respectively. The number of covariates used to adjust the multivariable model for the explored outcomes were decided based on the explored outcome of the sample size of to avoid overfitting. The specific covariates for multivariable adjustment were decided based on clinical meaning and their known association with outcomes including:

- Age;
- Patient past medical history – that was described by the presence of any comorbidities among the following ones (Chronic cardiovascular disease, Chronic pulmonary disease, Diabetes, Chronic renal disease, Cancer); and
- Patients current clinical illness severity by using SOFA score.

Results of the multivariate models were reported as β coefficient, Hazard Ratio (HR) with 95% CI (95% CI).

Daily differences up to 7 days since randomization in physiological variables between the study interventions (Sigh versus No Sigh) in the investigated physiological subgroups were assessed by using generalized estimating equation models account for repeated measures for subjects. Association between average physiological parameters within 7-d and study outcomes were performed by using Cox-proportional (i.e. 28-day mortality) and Fine & Gray models using mortality as a competitive event (i.e. successful extubation with more than 48 h free from reintubation at 28-day). Differences in ventilatory ratio between survivors and non-survivors were assessed by using Mann–Whitney U-test. Statistical analyses were performed with SAS 9.4 TS Levek 1M7 (2020 SAS Institute Inc., Cary, NC, USA) and R Studio 2002.07.1 (2009-2002Rstudio PBC).

Further details on methods are reported in the Supplemental material.

## Results

**Fig. 1:**
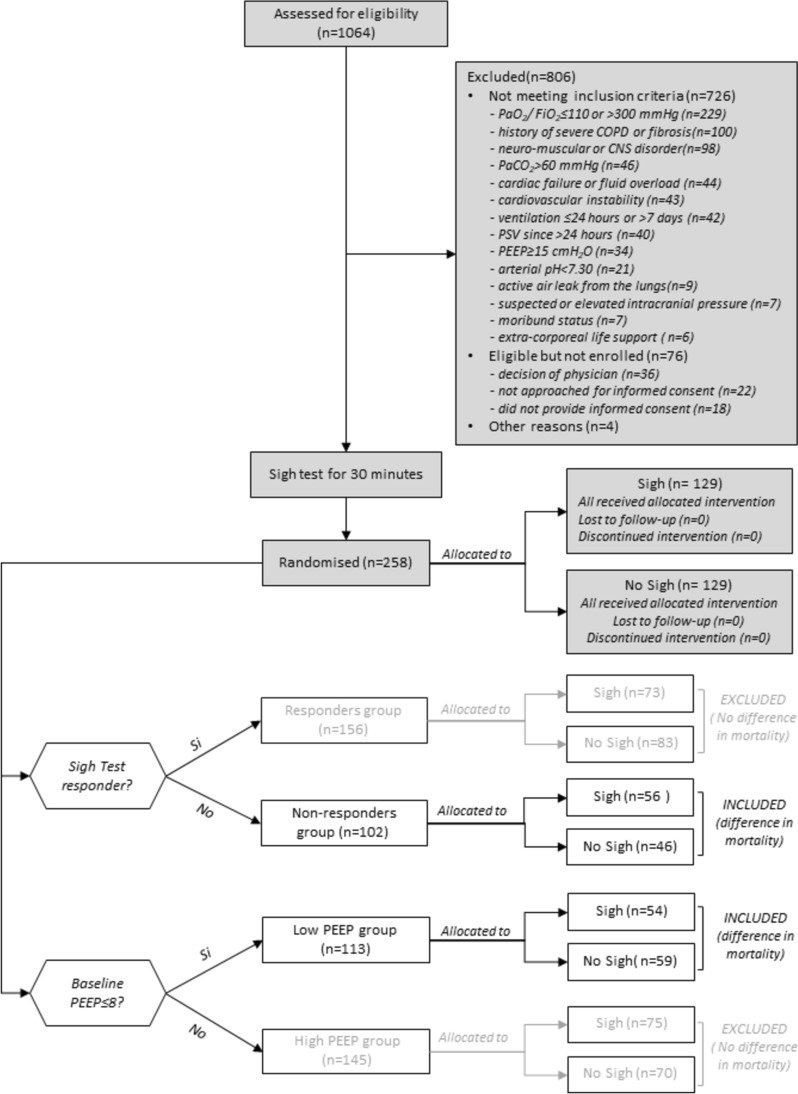
Patient selection criteria about the two investigated predefined physiological subgroups of patients included in the data analyses

**Fig. 2:**
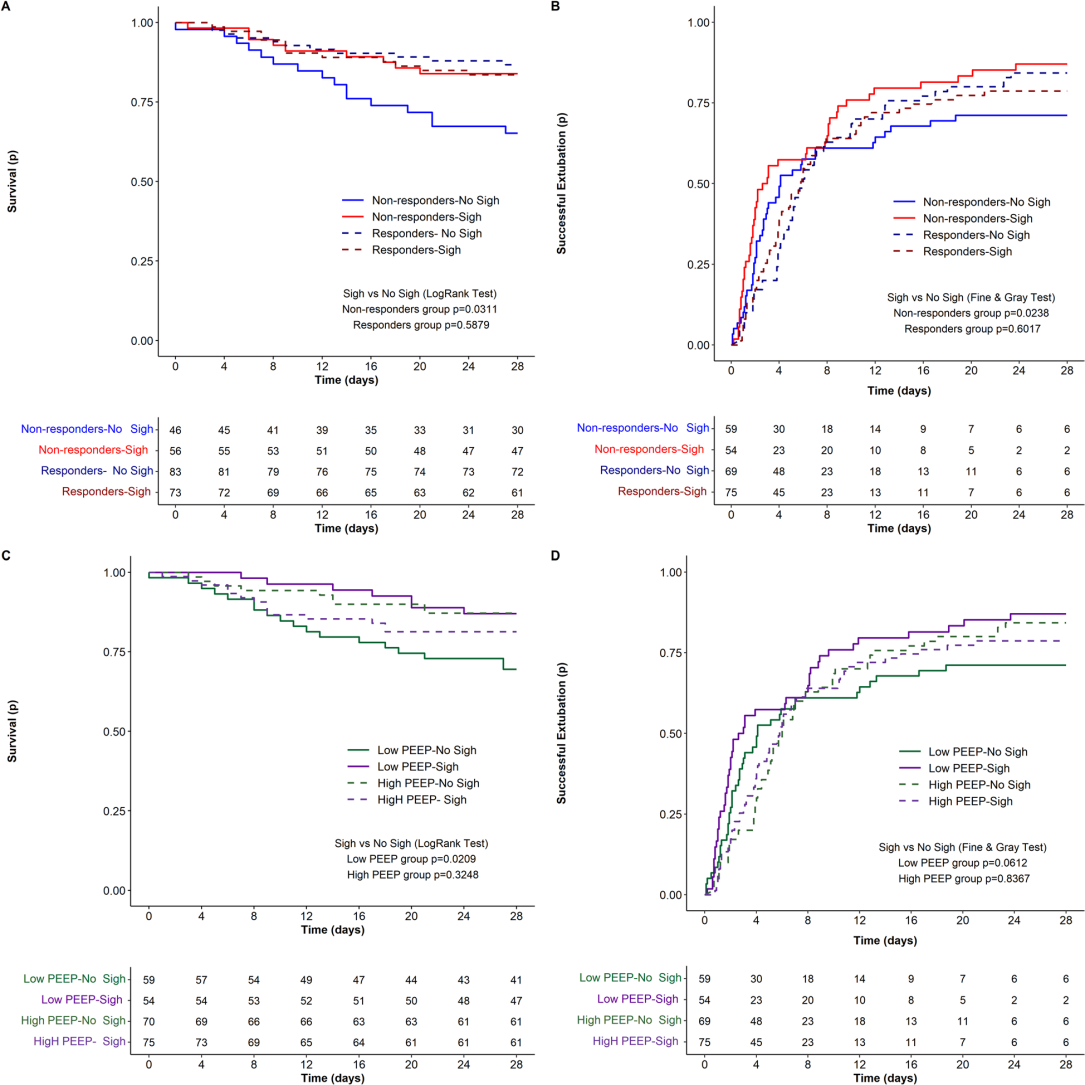
Death (**A**) and successful extubation (**B**) at 28-day follow-up in patients stratified by Sigh versus No Sigh in the Non-responders subgroup. Death (**C**) and successful extubation (**D**) at 28-day follow-up in patients stratified by Sigh versus No Sigh in the Low PEEP subgroup. (p) = probability. Number of patients at risk by groups are reported below each panel timeline

We explored differences in 28-day mortality and successful extubation in Sigh versus No Sigh treatments based on the oxygenation responsive to the baseline sigh test (responders versus non-responders) and to the exposure to different levels of PEEP (High versus Low PEEP). Patients included in the current analyses are reported in Fig. 1. Outcomes differed, in the 1) Oxygenation non-responder group (Fig. 2A, B, Supplemental Fig. 1A,B); and in the 2) Low PEEP group (Fig. 2C, D, Supplemental Fig. 1C,D). Therefore, we investigated physiological differences between Sigh versus No Sigh treatments in these 2 specific subgroups of patients.

### Baseline characteristics

#### Non-responders

Patients included in the Protection Trial fulfilling the criteria of oxygenation non-responder group were 102 out of 258 (40%). Fifty-six patients were randomized to Sigh (55%), while 46 to No Sigh (45%). Baseline characteristics of non-responders stratified by the randomization to Sigh were reported in Table 1. Only comorbidities differed between the study groups and were lower in the Sigh arm.

**Table 1:** Baseline characteristics of patients non-responders to Sigh oxygenation test stratified by the randomization to Sigh

	No Sigh (N = 46)	Sigh (N = 56)	p-value
*Demographics*			
Male—N (%)	38 (83)	43 (77)	0.4682
Age (years)—median (Q_1_–Q_3_)	65.5 (56.0–77.0)	66.5 (55.5–77.0)	0.9893
Heigh (cm)—median (Q_1_–Q_3_)	170 (162–175)	170 (165–178.5)	0.4985
Weight (Kg)—median (Q_1_–Q_3_)	80 (70–86)	79 (66–90)	0.9839
BMI—median (Q_1_–Q_3_)	26.43 (23.83–30.11)	25.98 (22.86–30.10)	0.5034
PBW^a^—median (Q_1_–Q_3_)	65.93 (56.90–70.45)	65.93 (60.95–73.62)	0.5910
*Past medical history—N (%)*			
N comorbidities—N (%)			
0	7 (16)	18 (32)	**0.0184**
1	10 (23)	14 (25)	
2	9 (21)	16 (29)	
≥ 3	18 (41)	8 (14)	
Charlson comorbidities			
Chronic cardiovascular disease^b^	32 (70)	32 (57)	0.1966
Chronic pulmonary disease	9 (20)	6 (11)	0.2092
Diabetes	10 (22)	9 (16)	0.4318
Chronic renal disease	11 (24)	6 (11)	0.0751
Cancer	8 (17)	4 (7)	0.1099
*Recent medical history—N(%)*			
Type of admission—N (%)			
Medical	36 (78)	43 (77)	0.8952
Surgical	10 (22)	13 (23)	
In-hospital LOS days^c^—median (Q_1_–Q_3_)	5.5 (3–9)	5 (3–7)	0.4287
ICU LOS days^c^—median (Q_1_–Q_3_)	3 (2–5)	3 (2–5)	0.8403
Intubation days^c^—median (Q_1_–Q_3_)	3 (1–5)	3 (2–4.5)	0.8403
*Systemic severity*			
SAPS II—median (Q1–Q3)	45 (33–55)	42 (30–53)	0.5624
SOFA—median (Q1–Q3)	8 (5–10)	8 (5–10)	0.6622
RASS—median (Q1–Q3)	−2 (−2 to 0)	−2 (−2 to 0)	0.4106
*Lung injury*			
Risk factors—N (%)			
Pneumonia	28 (61)	35 (63)	0.8661
Aspiration of gastric content	2 (4)	7 (13)	0.1795
Lung vasculitis	1 (2)	0 (0)	0.4510
Drowing	0 (0)	0 (0)	
Non-pulmonary sepsis	9 (20)	5 (9)	0.1203
Pancreatitis	1 (2)	1 (2)	> 0.9999
Severe burns	0 (0)	1 (2)	> 0.9999
TRALI	1 (2)	0 (0)	0.4510
Others	4 (9)	4 (7)	> 0.9999
Lung contusion + trauma	2 (4)	7 (13)	0.1795
Respiratory worsening—N (%)	40 (87)	47 (84)	0.6674
Evidence of pulmonary infiltrates—N (%)			
No	9 (20)	14 (25)	0.6464
Unilateral	14 (30)	19 (34)	
Bilateral	23 (50)	23 (41)	
*Ventilation clinical settings*			
PEEP (cmH_2_O)—median (Q_1_–Q_3_)	9 (8–10)	9.5 (8–12)	0.4997
PSV level (cmH_2_O)—median (Q_1_–Q_3_)	10 (8–12)	10 (8–12)	0.1843
RR (bpm)—median (Q_1_–Q_3_)	19 (15–22)	17 (14–21)	0.2523
PaO_2_ (mmHg)—median (Q_1_–Q_3_)	89 (73–101)	79 (73–95)	0.1771
FiO_2_—median (Q_1_-–Q_3_)	0.40 (0.35–0.40)	0.40 (0.35–0.45)	0.7363
PaO_2_/FiO_2_—median (Q_1_–Q_3_)	242 (198–270)	211 (190–257)	0.1904
PaCO_2_ (mmHg)—median (Q_1_–Q_3_)	44 (39–48)	45 (38–48)	0.8534
pH—median (Q_1_–Q_3_)	7.44 (7.39–7.48)	7.43 (7.40–7.46)	0.8877
*Sigh test*			
SpO_2_/FiO_2_, baseline—median (Q_1_–Q_3_)	269 (240–320)	271 (239–320)	0.7696
SpO_2_/FiO_2_, after 30 min—median (Q_1_–Q_3_)	258 (238–317)	264 (239–320)	0.6888
∆SpO_2_/FiO_2_, 30 min-baseline, absolute difference—median (Q_1_–Q_3_)	0 (−2.9 to 0)	0 (−2.9 to 0)	0.7218
∆SpO_2_/FiO_2_, 30 min-baseline, % difference—median (Q_1_–Q_3_)	0 (−0.01 to 0)	0 (−0.01 to 0)	0.6331
*Etiology*			
ARF etiology—N (%)			
Infectious pulmonary	28 (61)	40 (71)	0.1585
Infectious extrapulmonary	9 (18)	3 (5)	
Non-infectious pulmonary	3 (7)	6 (11)	
Non-infectious extrapulmonary	6 (13)	7 (13)	
ARF causes—N (%)			
Pneumonia	26 (57)	39 (70)	0.1702
Cardiac faiure	2 (4)	3 (5)	> 0.9999
Asthma	0 (0)	0 (0)	
ARDS	8 (17)	7 (13)	0.4876
COPD	2 (4)	0 (0)	0.2009
Unknown	3 (7)	2 (4)	0.6555
Other	12 (26)	14 (25)	0.9003

Statistically significant p-values are reported in bold

^a^PBW computed as ARDSNet Equation (Brower et al. [38])

^b^Myocardial Infarction, Congestive Heart Failure, Cerebrovascular Disease, Hypertension (at least one)

^c^Days at randomisation date

*ARF* acute respiratory failure, *BMI* body mass index, *ICU* intensive care unit, *LOS* length of stay, *PBW* predicted body weight, *PEEP* positive end-expiratory pressure, *PaCO*_*2*_ arterial carbon dioxide partial pressure, *PaO*_*2*_ arterial oxygen partial pressure, *PEEP* positive end-expiratory pressure, *pH* negative logarithm of hydrogen concentration, *PSV* pressure support ventilation, *RR* respiratory rate, *SAPS* simplified acute physiology score, *SOFA* sequential organ failure assessment, *TRALI* transfusion related acute lung injury

#### Low PEEP group

Patients included in the Protection Trial fulfilling the criteria of Low PEEP (PEEP levels ≤ 8cmH_2_O—median PEEP level of the original study) were 113 out of 258 (44%). Fifty-four patients were randomized to Sigh (48%), while 59 to No Sigh (52%). Baseline characteristics of Low PEEP patients stratified by the randomization to sigh were reported in Table 2. No differences were reported between the two arms.

**Table 2:** Baseline characteristics of patients exposed to Low PEEP stratified by the randomization to Sigh

	No Sigh (N = 59)	Sigh (N = 54)	p-value
*Demographics*			
Male—N (%)	45 (76)	35 (65)	0.1809
Age (years)—median (Q_1_–Q_3_)	65.0 (58.0–75.0)	67.0 (57.0–79.0)	0.4444
Heigh (cm)—median (Q_1_–Q_3_)	170 (160–178)	170 (162–175)	0.6708
Weight (Kg)—median (Q_1_–Q_3_)	72 (64–85)	76 (63–85)	0.8584
BMI—median (Q_1_–Q_3_)	25.66 (22.86–27.78)	25.67 (22.77–28.73)	0.8586
PBW^a^—median (Q_1_–Q_3_)	65.93 (56.88–73.17)	65.93 (54.19–70.45)	0.6074
*Past medical history—N (%)*			
N comorbidities—N (%)^b^			
0	7 (12)	14 (26)	0.1388
1	15 (26)	16 (30)	
2	15 (26)	13 (24)	
≥ 3	21 (36)	11 (20)	
Charlson comorbidities			
Chronic cardiovascular disease^c^	39 (66)	29 (54)	0.1787
Chronic pulmonary disease	17 (29)	9 (17)	0.1254
Diabetes	13 (22)	9 (17)	0.4443
Chronic renal disease	12 (20)	6 (11)	0.1806
Cancer	9 (15)	5 (9)	0.3339
*Recent medical history—N(%)*			
Type of admission—N (%)			
Medical	40 (68)	34 (63)	0.5893
Surgical	19 (32)	20 (37)	
In-hospital LOS days^d^—median (Q_1_–Q_3_)	5 (3–12)	4.5 (3–8)	0.3472
ICU LOS days^d^—median (Q_1_–Q_3_)	3 (1–4)	3 (2–4)	0.8042
Intubation days^d^- median (Q_1_–Q_3_)	2 (1–4)	2 (1–4)	0.7132
*Systemic severity*			
SAPS II—median (Q1–Q3)	42 (30–56)	42 (34–55)	0.6713
SOFA—median (Q1–Q3)	8 (6–9)	8 (5–10)	0.9540
RASS—median (Q1–Q3)	−2 (−2 to −1)	−2 (−2 to 0)	0.1897
*Lung injury*			
Risk factors—N (%)			
Pneumonia	31 (52)	28 (52)	0.9415
Aspiration of gastric content	4 (7)	5 (9)	0.7348
Lung vasculitis	1 (2)	0 (0)	> 0.9999
Drowing	0 (0)	0 (0)	
Non-pulmonary sepsis	14 (24)	12 (22)	0.8493
Pancreatitis	2 (3)	1 (2)	> 0.9999
Severe burns	0 (0)	0 (0)	
TRALI	1 (2)	0 (0)	0.4510
Others	6 (10)	4 (7)	0.7450
Lung contusion + trauma	3 (5)	5 (9)	0.4761
Respiratory worsening—N (%)	51 (86)	34 (63)	**0.0039**
Evidence of pulmonary infiltrates—N (%)			
No	13 (22)	20 (37)	0.0953
Unilateral	19 (32)	19 (35)	
Bilateral	27 (46)	15 (28)	
*Ventilation clinical settings*			
PEEP (cmH_2_O)—median (Q_1_–Q_3_)	8 (6–8)	8 (6–8)	0.9242
PSV level (cmH_2_O)—median (Q_1_–Q_3_)	10 (8–12)	8 (8–10)	0.4287
RR (bmp)—median (Q_1_–Q_3_)	18 (15–22)	18 (15–22)	0.5557
PaO_2_ (mmHg)—median (Q_1_–Q_3_)	92 (75–108)	83 (73–96)	0.1090
FiO_2_—median (Q_1_–Q_3_)	0.40 (0.35–0.50)	0.40 (0.30–0.40)	0.5180
PaO_2_/FiO_2_—median (Q_1_–Q_3_)	238 (203–270)	233 (200–258)	0.6797
PaCO_2_ (mmHg)—median (Q_1_–Q_3_)	43 (39–46)	42 (36–47)	0.4803
pH—median (Q_1_–Q_3_)	7.43 (7.38–7.46)	7.43 (7.39–7.47)	0.8948
*Sigh test*			
SpO_2_/FiO_2_, baseline—median (Q_1_–Q_3_)	248 (233–317)	273 (238–320)	0.2034
SpO_2_/FiO_2_, after 30 min—median (Q_1_–Q_3_)	250 (233–323)	279 (240–320)	0.1207
∆SpO_2_/FiO_2_, 30 min-baseline, absolute difference—median (Q_1_–Q_3_)	2.5 (0.0–6.7)	2.3 (0.0–5.7)	0.4074
∆SpO_2_/FiO_2_, 30 min-baseline, % difference—median (Q_1_–Q_3_)	0.01 (0.00–0.20)	0.01 (0.00–0.20)	0.6114
*Etiology*			
ARF etiology—N (%)			
Infectious pulmonary	32 (54)	29 (54)	0.5474
Infectious extrapulmonary	14 (24)	8 (15)	
Non-infectious pulmonary	5 (9)	7 (13)	
Non-infectious extrapulmonary	8 (14)	10 (19)	
ARF causes—N (%)			
Pneumonia	33 (56)	32 (59)	0.7208
Cardiac faiure	3 (5)	4 (7)	0.7077
Asthma	0 (0)	1 (2)	0.4779
ARDS	3 (5)	6 (11)	0.4876
COPD	1 (2)	1 (2)	> 0.9999
Unknown	6 (10)	4 (7)	0.7450
Other	18 (31)	16 (30)	> 0.9999

Statistically significant p-values are reported in bold

^a^PBW computed as ARDS Net Equation (Brower et al. [38])

^b^One missing datum

^c^Myocardial Infarction, Congestive Heart Failure, Cerebrovascular Disease, Hypertension (at least one)

^d^Days at randomization date

*ARF* acute respiratory failure, *BMI* body mass index, *ICU* intensive care unit, *LOS* length of stay, *PBW* predicted body weight, *PEEP* positive end-expiratory pressure, *PaCO*_*2*_ arterial carbon dioxide partial pressure, *PaO*_*2*_ arterial oxygen partial pressure, *PEEP* positive end-expiratory pressure, *pH* negative logarithm of hydrogen concentration, *PSV* pressure support ventilation, *RR* respiratory rate, *SAPS* simplified acute physiology score, *SOFA* sequential organ failure assessment, *TRALI* transfusion related acute lung injury

### Clinical outcomes

#### Non-responders

We evaluated differences in outcomes between the study arms. In the Sigh treatment, 28-day mortality was lower, proportion of patients successfully extubated was higher and duration of ventilator free days was longer as compared with No Sigh treatment (Supplemental Table 1).

We explored differences in mortality by time-to-event analysis between the study arms. We observed that mortality over 28-day follow-up was significantly lower in the Sigh versus No Sigh arm (log-rank p = 0.031) (Fig. 2A, Supplemental Fig. 1A). After adjusting the multivariate model for clinically meaningful variables (i.e.age, comorbidities and SOFA score) the use of Sigh was consistently associated with a decreased mortality (HR 0.40; 95% CI 0.17–0.92; p = 0.030) (Table 3).

**Table 3:** Multivariate Cox Model on death and Fine & Gray Model on successful extubation (mortality as a Competitive Event) at 28 days in the non-responder group

Parameter	Beta	HR	95% CI	p-value
*28-day mortality*				
Age (years)	0.03549	1.036	(1.001: 1.073)	**0.0452**
Comorbidities^a^ (vs No)	0.22865	1.257	(0.350; 4.510)	0.7258
SOFA (unit)	0.12139	1.129	(1.008; 1.264)	**0.0352**
Sigh (vs No Sigh)	−0.92874	0.395	(0.171; 0.915)	**0.0302**
*Successful extubation at 28-day follow-up*				
Age (years)	−0.01680	0.983	(0.965; 1.002)	0.0731
Comorbidities^a^ (vs No)	0.30000	1.350	(0.762; 2.393)	0.3044
SOFA (unit)	0.01606	1.016	(0.963; 1.072)	0.5573
Sigh (vs No Sigh)	0.57642	1.780	(1.080; 2.933)	**0.0238**

Statistically significant p-values are reported in bold

^a^Any comorbidities

*SOFA* sequential organ assessment failure

As a second clinical outcome, we investigated differences in the proportion of patients successfully extubated by competing risk analyses. We observed that the proportion of patients with a successful extubation at 28-day follow-up was higher in the Sigh versus No Sigh arm (Fine & Gray p = 0.024) (Fig. 2B, Supplemental Fig. 1B). After adjusting the model for clinically meaningful variables the use of Sigh was consistently associated with an increased successful extubation (HR 1.78; 95% CI 1.08–2.93; p = 0.024) (Table 3).

Of note, Sigh was not associated with differences in 28-day mortality (16% vs. 13%, p = 0.575) and successful extubation (81% vs. 85%, p = 0.6017) in patients with positive response to baseline sigh test (Fig. 2A, B).

#### Low PEEP group

We evaluated differences in outcomes between the study arms. In the Sigh arm, 28-day mortality was lower, and proportion of patients successfully extubated was higher in survivors, while duration of ventilator free days did not differ as compared with No Sigh treatment (Supplemental Table 2). We explored differences in mortality by time-to-event analyses between the study arms. We observed that mortality over 28-day follow-up was significantly lower in the Sigh versus No Sigh arm (log-rank p = 0.021) (Fig. 2C, Supplemental Fig. 1C). After adjusting the multivariate model for clinically meaningful variables the use of sigh was consistently associated with a decreased mortality (HR 0.26; 95% CI 0.10–0.68; p = 0.005) (Table 4).

**Table 4:** Multivariate Cox Model on death and Fine & Gray Model on successful extubation (mortality as a Competitive Event) at 28 days in the Low PEEP group

Parameter	Beta	HR	95% CI	p-value
*28-day mortality*				
Age (years)	0.04996	1.051	(1.013; 1.091)	**0.0082**
Comorbidities^a^ (vs No)	0.40393	1.498	(0.344; 6.521)	0.5905
SOFA (unit)	−0.00330	0.997	(0.863; 1.152)	0.9643
Sigh (vs No Sigh)	−1.33381	0.263	(0.103; 0.675)	**0.0054**
*Successful extubation at 28-day follow-up*				
Age (years)	−0.01253	0.988	(0.972; 1.004)	0.1332
Comorbidities^a^ (vs No)	0.12195	1.130	(0.620; 2.058)	0.6903
SOFA (unit)	−0.02568	0.975	(0.916; 1.037)	0.4169
Sigh (vs No Sigh)	0.55795	1.747	(1.146; 2.663)	**0.0095**

Statistically significant p-values are reported in bold

^a^Any comorbidities

*SOFA* sequential organ failure assessment

As a second clinical outcome, we investigated differences in the proportion of patients successfully extubated by competing risk analyses. We observed that the proportion of patients successfully extubated over 28-day follow-up trended higher in the Sigh arm as compared with the No Sigh (Fine & Gray p = 0.061) (Fig. 2D, Supplemental Fig. 1D). After adjusting the model for clinically meaningful variables the use of sigh was associated with an increased successful extubation (HR 1.75; 95% CI 1.15–2.66; p = 0.010) (Table 4).

Of note, Sigh treatment was not associated with differences in 28-day mortality (19% versus 13%, p = 0.339) and successful extubation (79% vs. 84%, p = 0.8367) in patients with clinical PEEP > 8cmH_2_O (Fig. 2C, D).

Exploratory differences in the proportion of 28-day mortality and successful extubation by competing risk analyses between predefined physiological subgroups exposed or not exposed to SIGH are reported in Supplemental Table 3.

### Ventilatory parameters at 7 days

#### Non-responders

**Fig. 3:**
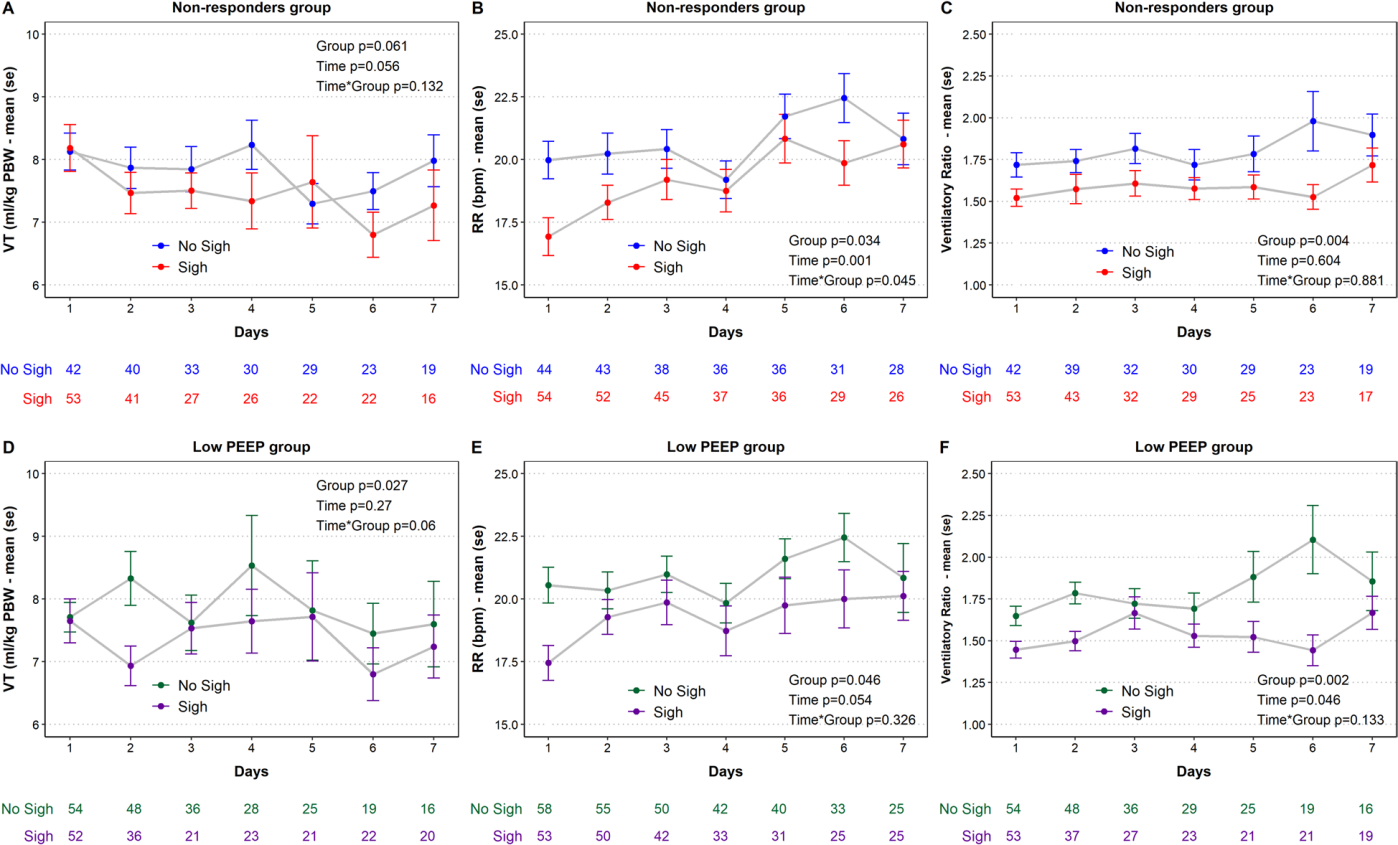
Vt/PBW (**A**), RR (**B**) and Ventilatory ratio (**C**) differences over 7-day follow up since randomization between Sigh versus No Sigh in the Non-responders group. Vt/PBW (**D**), RR (**E**) and Ventilatory ratio (**F**) differences over 7-day follow up since randomization between Sigh versus No Sigh in the Low PEEP group. *PBW* predicted body weight, *PEEP* positive end-expiratory pressure, *RR* respiratory rate, *SE* standard error, *VT* tidal volume

We explored longitudinal physiological daily differences between arms up to 7 days since randomization. Ventilator settings did not differ between the groups (i.e.PSV, PEEP and FiO_2_). While oxygenation did not change between the groups, PaCO_2_ trended to lower levels in the Sigh arm (Supplemental Fig. 2). Despite minute ventilation did not significantly decrease in the Sigh arm (Supplemental Fig. 2), Vt/PBW trended to lower levels while RR decreased significantly in the Sigh arm (Fig. 3A, B). Therefore, we explored differences in proxies of pulmonary dead space between the 2 groups. We observed that both standardized minute ventilation (Supplemental Fig. 2) and ventilatory ratio were significantly lower in the Sigh versus No Sigh arm (Fig. 3C).

#### Low PEEP group

We explored longitudinal physiological daily differences between arms up to 7 days since randomization. Ventilator settings did not differ between the groups (i.e.PSV, PEEP and FiO_2_) and so did not gas exchange. Interestingly, minute ventilation decreased significantly in the Sigh treatment (Supplemental Fig. 3). This was led by both a decrease in Vt/PBW and lower respiratory rate as compared with the Sigh treatment (Fig. 3D, E). We explored differences in proxies of pulmonary dead space between the 2 groups. We observed that both standardized minute ventilation (Supplemental Fig. 3) and ventilatory ratio (Fig. 3F) were significantly lower in the Sigh versus No Sigh arm.

Comprehensive data about differences over 7-day follow-up about ventilatory and physiological parameters between Sigh versus No Sigh in both physiological subgroups are reported in the Supplemental material.

### Association between ventilatory parameters and outcomes

#### Non-responders

While different ventilatory parameters during 7-day follow-up were correlated with successful extubation, only RR and ventilatory ratio were both positively correlated with 28-day mortality (Table 5, Supplemental Fig. 4).

**Table 5:** Association between 7-day average levels of physiological ventilatory variables with death and successful extubation at 28 day follow-up in both physiological subgroups

Physiological parameters	Non-responders			Low PEEP		
	HR (Sigh vs No Sigh)	95% CI	p-value	HR (Sigh vs No Sigh)	95% CI	p-value
*28-day mortality*						
Vt/PBW, mL/kg	0.972	(0.758; 1.246)	0.8227	1.017	(0.849; 1.217)	0.8571
RR, breaths/min	1.123	(1.011; 1.246)	**0.0296**	1.117	(1.015; 1.229)	**0.0236**
VR	2.604	(1.002; 6.765)	**0.0495**	4.015	(1.682; 9.585)	**0.0017**
PEEP, cmH_2_O	1.102	(0.927; 1.309)	0.2722	1.103	(0.890; 1.368)	0.3698
PSV level, cmH_2_O	1.089	(0.96; 1.236)	0.1843	1.046	(0.934; 1.172)	0.4321
PaO_2_/FiO_2_, mmHg	0.994	(0.988; 1.001)	0.1105	0.997	(0.990; 1.004)	0.3816
PaCO_2_, mmHg	1.014	(0.967; 1.063)	0.5712	1.043	(1.000; 1.089)	0.0524
Ph	0.022	(0.000; 948.673)	0.4839	0.003	(0.000; 24.513)	0.2057
p0.1, cmH_2_O	0.826	(0.543; 1.256))	0.3711	0.923	(0.636; 1.34)	0.6751
Mve, L/min	1.137	(0.946; 1.367)	0.1713	1.139	(0.950; 1.367)	0.1606
stMve, L/min	1.141	(0.991; 1.314)	0.0660	1.162	(1.018; 1.327)	**0.0261**
*Successful extubation at 28-day follow-up*						
Vt/PBW, mL/kg	0.942	(0.816; 1.088)	0.4159	0.995	(0.922; 1.073)	0.8906
RR, breaths/min	0.999	(0.917; 1.089)	0.9867	0.941	(0.893; 0.992)	**0.0227**
VR	0.343	(0.173; 0.681)	**0.0022**	0.313	(0.167; 0.584)	**0.0003**
PEEP, cmH_2_O	0.834	(0.744; 0.934)	**0.0018**	0.8	(0.695; 0.922)	**0.0021**
PSV level, cmH_2_O	0.902	(0.843; 0.964)	**0.0026**	0.919	(0.853; 0.99)	**0.0267**
PaO_2_/FiO_2_, mmHg	1.005	(1.001; 1.010)	**0.0240**	1.004	(1.000; 1.009)	**0.0417**
PaCO_2_, mmHg	0.981	(0.956; 1.007)	0.1459	0.974	(0.950; 0.998)	**0.0319**
Ph	0.254	(0.001; 106.923)	0.6565	33.765	(0.292; 3898.015)	0.1463
p0.1, cmH_2O_	1.25	(1.049; 1.489)	**0.0126**	0.991	(0.808; 1.215)	0.9296
Mve, L/min	0.874	(0.770; 0.993)	**0.0386**	0.864	(0.779; 0.957)	**0.0053**
stMve, L/min	0.883	(0.805; 0.968)	**0.0083**	0.841	(0.769; 0.920)	**0.0002**

Statistically significant p-values are reported in bold

*PBW* predicted body weight, *FiO*_*2*_ inspiratory oxygen fraction, *Mve* expiratory minute ventilation, *PaCO*_*2*_ arterial carbon dioxide partial pressure, *PaO*_*2*_ arterial oxygen partial pressure, *PEEP* positive end-expiratory pressure, *pH* negative logarithm of hydrogen concentration, *PSV* pressure support ventilation, *P0.1* occlusion pressure at 100 ms, *RR* respiratory rate, *stMve* standardized expiratory minute ventilation, *VR* ventilatory ratio, *Vt* tidal volume

#### Low PEEP group

While different ventilatory parameters during 7-day follow-up were correlated with successful extubation, only respiratory rate, ventilatory ratio and standardized minute ventilation were both positively correlated with 28-day mortality (Table 5, Supplemental Fig. 4).

## Discussion

In this secondary analysis of the PROTECTION randomized controlled trial -assessing the feasibility of sigh during pressure support ventilation- we aimed at reducing patient heterogeneity by investigating the role of sigh breathing in different predefined physiological subgroups of patients. This was based on 1. the oxygenation response to a 30-min sigh test before randomization, and 2. different levels of set PEEP.

The main findings of our investigation include the following ones:

- sigh was not associated with differences in 28-day mortality in responders and in patients with clinical set PEEP > 8cmH_2_O; surprisingly, 28-d mortality was significantly lower in the Sigh versus No Sigh arm in non-responders, and in patients exposed to Low PEEP levels; this was further confirmed after adjustment in multivariable models;
- in non-responders, successful extubation was significantly higher in the Sigh versus No Sigh arm, and similarly, a trend was observed in the Low PEEP group; this was confirmed in both subgroups after adjustment in multivariable models;
- daily Vt/PBW and respiratory rate levels were lower in the Sigh versus No Sigh arm up to 7-day follow-up in both subgroups;
- pulmonary dead space and ventilation-perfusion mismatch -estimated by using ventilatory ratio- was lower in the Sigh versus No Sigh arm at 7-day follow-up in both subgroups;
- ventilatory ratio was the only parameter associated with both 28-day mortality and successful extubation in both predefined physiological subgroups of patients (i.e. Non-responders and Low PEEP groups).

In these prespecified secondary analyses of the PROTECTION trial we observed that the use of sigh was associated with favourable outcomes. So far, the only RCT exploring differences on outcome using sigh during controlled mechanical ventilation—the SiVent study—suggested a promising beneficial role on outcome in the treatment arm with SIGH [8]. In our analysis, we confirmed the positive association with the use of sigh during PSV and a better outcome in 2 different predefined physiological subgroups. This was confirmed after multivariable adjustment with robust clinical variables known to have an impact on outcome in patients with respiratory failure undergoing mechanical ventilation: age [18]; patient past clinical history (i.e. comorbidities) [19]; and clinical illness severity estimated by the severity of organ failures [20]. Although these findings are exploratory and preliminary, they question whether a periodic brief recruitment manoeuvre during assisted mechanical ventilation may contribute somehow to the optimization of the pulmonary function and consequently may influence outcomes. The recent ESICM guidelines suggestions against routine use of brief high-pressure RM to reduce mortality in patients of ARDS consider a RM ≥ 35 cmH_2_O for less than a minute. In our setting, a RM pressure of 30 cmH_2_O for a duration of only 3 s may probably and unlikely result in complications, including barotrauma and hemodynamic instability [21].

We further evaluated daily differences in the levels of ventilatory variables over time (7 days after randomization) between Sigh versus No Sigh arm to infer on mechanisms that may support differences in major outcomes between predefined physiological subgroups.

The physiological benefit of sigh during controlled mechanical ventilation in ARDS is widely recognized [7, 22, 23]. During spontaneous breathing sigh promotes variability of tidal and minute volume ventilation in healthy infants [24]. Tidal volume variability is suggested to improve patient-ventilator asynchronies [25], which is associated with better outcomes [26]. Furthermore, in patients with respiratory failure a low tidal volume variability seems to be associated with the presence of dyspnoea as compared to healthy subjects [27]. Sigh was described to improve both respiratory mechanics—by increasing EELI and consequently the respiratory system compliance—and gas exchange during PSV [10]. Furthermore, sigh makes the regional distribution of the tidal ventilation more homogeneous [11]. In our analyses we observed a decrease in pulmonary dead space in the Sigh arm in both physiological subgroups. This was observed by a lower standardized minute ventilation and a lower ventilatory ratio. Further, this was achieved by decreasing PaCO_2_ levels over time in the Sigh versus No Sigh arm—although not significantly—and by both a decrease in Vt/PBW and respiratory rate. The beneficial role of sigh on decreasing the pulmonary dead space may suggest a potential contribution in the decrease of death in our population [28]. Both increasing levels of standardized minute ventilation [9, 29] and ventilatory ratio [30] are associated with worse outcomes in patients with ARDS. Furthermore, the decrease in wasted ventilation may suggest an improved homogeneous regional ventilation leading to a better optimization of the ventilation perfusion matching [11] which is associated with a better outcome [31]. These findings are potentially of high clinical relevance during the ventilatory management. The decision on setting sigh during pressure support ventilation may not be driven only by an improvement of oxygenation—as it was performed in the PROTECTION original trial. Sigh may be set during PSV by assessing the response on the decrease in physiologic deadspace—that can be easily estimated at bedside by using standardized minute ventilation or ventilatory ratio. We may speculate that patients exposed to Sigh may show a better outcome in the presence of a decreased ventilatory ratio as compared with No Sigh. This is in line with the superior role of CO_2_ clearing—as compared to oxygenation improvement—in predicting a lower mortality rate in ARDS patients as a consequent effect of lung recruitment after prone-positioning [32]. Of note, in both our physiological subgroups, ventilatory ratio was the only variable positively associated with both 28-day mortality and successful extubation.

Another key physiological finding is the enhancement of protective ventilation by Sigh treatment. This may contribute to the beneficial role of SIGH on outcome by decreasing the intensity of ventilation [29]. The decrease of tidal volume was recently suggested to protect the lung from the patient self-inflicted lung injury [33, 34]. Furthermore, the lower respiratory rate seems to reduce the risk of lung injury [35] and may have an independent contribution on outcome in patients with ARDS [36].

Taking all these findings together, we may question on the reasons why sigh is beneficial when the patients are non-responders to the Sigh test as compared with responders. In non-responders to the 30-min Sigh test, Sigh does not seem to provide a significant benefit on oxygenation either during the Sigh test or after randomization at 7 days, suggesting that the main mechanism of Sigh breathing may not be immediate lung recruitment during PSV. The beneficial role of Sigh after randomization may be explained by a decrease of physiologic dead-space and therefore of ventilation intensity (i.e. tidal volume and respiratory rate). However, it is also possible that the repeated sighs allow lung volume to remain stable instead of gradually decreasing over hours without sighs, which may not be captured by the immediate response to the Sight test. Patients exposed to Low PEEP as compared with High PEEP may also experience a much better maintenance of lung volume over time. In patients exposed to Low PEEP, two conclusions may be inferred: 1. insufficient PEEP was used, as mortality was higher as compared with the High PEEP group; or 2. low PEEP should be used with intermittent sighs. Interestingly, even in this setting, the use of Sigh after randomization in the Low PEEP group does not seem to play a relevant role on optimizing oxygenation. It may also act by decreasing physiologic dead-space and ventilation intensity.

Strengths of the study include the secondary analysis on prespecified subgroups from a RCT. We have daily granular information on physiological parameters in all patients. The physiological findings in the 2 different subgroups are similar suggesting that differences on outcomes driven by Sigh treatment may include an optimization in ventilation perfusion matching, as seen by VR modulation. This analysis has also some limitations. We cannot infer on causal-effect interpretation. However, we adjusted our analyses for major predictors of clinical outcomes. As the sample size is limited, our results are exploratory and hypothesis generating and need further investigation. However, this is the only RCT performed in patients with AHRF exploring the role of Sigh treatment and investigating its role on the heterogeneity of treatment response in predefined physiological subgroups.

## Conclusions

In conclusions, sigh breathing during PSV was independently associated with better outcomes as compared with No Sigh ventilation in specific physiological subgroups of patients with AHRF. The findings in non-responders were not expected and require further exploration. The findings in the low PEEP group may indicate that insufficient PEEP was used or that low PEEP should be used with intermittent sighs [37].

Sigh treatment showed lower Vt/PBW and respiratory rate despite similar/lower CO_2_ levels leading to better ventilation/perfusion mismatch as compared with No Sigh. This was independently associated with major outcomes. Responsivity to brief recruitment manoeuvres during PSV may be investigated by the decrease of wasted ventilation (i.e. decreased pulmonary dead space)—that can be easily evaluated at bedside. This may introduce the concept of dead space responder as compared with oxygenation responder to Sigh.

These exploratory findings may help to identify distinct physiological subgroups of AHRF undergoing PSV who may benefit of Sigh breathing.

## Supplementary Information

Supplementary material 1.

## Data Availability

Data are available upon reasonable request to the Corresponding Author.

## Abbreviations

AHRF: Acute hypoxemic respiratory failure
ARDS: Acute respiratory distress syndrome
ARF: Acute respiratory failure
BMI: Body mass index
COPD: Chronic obstructive pulmonary disease
ESICM: European Society of Intensive Care Medicine
FiO_2_: Inspiratory oxygen fraction
ICU: Intensive care unit
LOS: Length of stay
Mve: Expiratory minute ventilation
PaCO_2_: Arterial carbon dioxide partial pressure
PaO_2_: Arterial oxygen partial pressure
PEEP: Positive end-expiratory pressure
pH: Negative logarithm of hydrogen concentration
PLUG: Pleural Pressure Working Group
PSV: Pressure support ventilation
P0.1: Occlusion pressure at 100 ms
Q: Quartile
RASS: Richmond Agitation-Sedation Scale
RM: Recruitment maneuver
RR: Respiratory rate
SAPS: Simplified Acute Physiology Score
SBT: Spontaneous breathing trial
SOFA: Sequential organ failure assessement
SpO_2_: Peripheral oxygen saturation
stMve: Standardized expiratory minute ventilation
TRALI: Transfusion related acute lung injury
VR: Ventilatory ratio
Vt: Tidal volume
