# Supplementary material for: Heterogeneous impact of Sighs on mortality in patients with acute hypoxemic respiratory failure: insights from the PROTECTION study
Source: Ann Intensive Care. 2024 Oct 5;14:153. doi: 10.1186/s13613-024-01385-0 (PMC11456003; doi:10.1186/s13613-024-01385-0)
Supplement: Supplementary file 1 — Supplementary material 1. [file 13613_2024_1385_MOESM1_ESM.docx]

**Supplemental material**

**Heterogeneous impact of Sighs on mortality in Patients with Acute Hypoxemic Respiratory Failure – *Insights from the PROTECTION study.***

**Authors:** Emanuele Rezoagli^1,2^, Carla Fornari^3^, Roberto Fumagalli^1,4^, Giacomo Grasselli^5,6^, Carlo Alberto Volta^7,8^, Paolo Navalesi^9,10^, Rihard Knafelj^11^, Laurent Brochard^12,13^, Antonio Pesenti^5,6^, Tommaso Mauri^5,6^*, Giuseppe Foti^1,2^*, for the Pleural Pressure Working Group (PLUG)

**Collaborators:** Riccardo Colombo MD (Department of Anesthesiology and Intensive Care, ASST Fatebenefratelli Sacco, Milan, Italy), Andrea Cortegiani MD (Section of Anesthesia, Analgesia, Intensive Care and Emergency, Department of Surgical, Oncological and Oral Science, Policlinico Paolo Giaccone, University of Palermo, Palermo, Italy), Jian-Xin Zhou MD (Department of Critical Care Medicine, Beijing Tiantan Hospital, Capital Medical University, Beijing, China), Rocco D’Andrea MD (Department of Anesthesiology, Intensive Care and Transplants, University Hospital St. Orsola-Malpighi, Bologna, Italy), Italo Calamai MD (Anesthesia and Intensive Care Unit AUSL Toscana Centro, Ospedale San Giuseppe, Empoli, Italy), Ánxela Vidal González MD (the Hospital Universitario Fundación Jiménez Díaz de Madrid, Madrid, Spain), Oriol Roca MD (Critical Care Department, Vall d’Hebron University Hospital, Vall d’Hebron Research Institute, Universitat Autònoma de Barcelona, Barcelona, Spain; the Ciber Enfermedades Respiratorias (CibeRes), Instituto de Salud Carlos III, Madrid, Spain), Domenico Luca Grieco MD (the Department of Anesthesiology and Intensive Care Medicine, Catholic University of the Sacred Heart, IRCCS Fondazione Policlinico A. Gemelli, Rome, Italy), Tomas Jovaisa MD (the Critical Care Service, Anaes- thetics Division, Barking Havering and Redbridge University Hospitals NHS Trust, London, United Kingdom), Dimitrios Bampalis MD (the Intensive Care Unit, Larissa General Hospital, Larissa, Greece), Tobias Becher MD (the Klinik für Anästhesiologie und Operative Intensivmedizin, Universitätsklinikum Schleswig-Holstein, Campus Kiel, Kiel, Germany), Denise Battaglini MD (Department of Surgical Sciences and Integrated Diagnostics, University of Genoa, Genoa, Italy; Anesthesia and Intensive Care, San Martino Policlinico Hospital, IRCCS for Oncology and Neurosciences, Genoa, Italy), Huiqing Ge MD (Sir Run Run Shaw Hospital, Zhejiang University School of Medicine, Hangzhou, Chin), Mariana Luz MD (Intensive Care Department, Hospital da Mulher, Salvador, Bahia, Brazil; the Intensive Care Department, Hospital Universitário Professor Edgard Santos, Universidade Federal da Bahia, Salvador, Bahia, Brazil), Jean-Michel Constantin MD (Sorbonne University, GRC 29, AP-HP, DMU DREAM, Department of Anesthesiology and Critical Care, Pitié-Salpêtrière Hospital, Paris, France), Marco Ranieri MD (Department of Anesthesiology, Intensive Care and Transplants, University Hospital St. Orsola-Malpighi, Bologna, Italy), Claude Guerin MD (Médecine Intensive-Réanimation Groupement Hospitalier Edouard Herriot, Université de Lyon Faculté de Médecine Lyon-Est, Lyon, France), Jordi Mancebo MD (the Servei de Medicina Intensiva, Hospital de la Santa Creu i Sant Pau, Universitat Autònoma de Barcelona (UAB), Barcelona, Spain), Paolo Pelosi MD (Department of Surgical Sciences and Integrated Diagnostics, University of Genoa, Genoa, Italy; Anesthesia and Intensive Care, San Martino Policli- nico Hospital, IRCCS for Oncology and Neurosciences, Genoa, Italy)

**Affiliations**

^1^School of Medicine and Surgery, University of Milano-Bicocca, Monza, Italy

^2^Department of Emergency and Intensive Care, Fondazione IRCCS San Gerardo dei Tintori Monza, Italy

^3^Research Centre on Public Health, University of Milano-Bicocca, Monza, Italy

^4^Department of Anesthesia and Intensive Care Medicine, ASST Grande Ospedale Metropolitano Niguarda, Milan, Italy

^5^Department of Pathophysiology and Transplantation, University of Milan, Milan, Italy

^6^Department of Anesthesia, Intensive Care and Emergency, Fondazione IRCCS Ca’ Granda Ospedale Maggiore Policlinico, Milan, Italy

^7^Department of Translational Medicine, University of Ferrara, Italy.

^8^Anesthesia and Intensive Care Unit, Emergency Department, Azienda Ospedaliera Universitaria di Ferrara, Italy

^9^Institute of Anesthesia and Intensive Care, Padua University Hospital, Padova, Italy

^10^Department of Medicine (DIMED), University of Padua, Padova (PD) Italy

^11^Center for Internal Intensive Medicine, University Medical Center Ljubljana, Ljubljana, Slovenia

^12^Interdepartmental Division of Critical Care Medicine, University of Toronto, Toronto, Canada

^13^Keenan Research Centre for Biomedical Science, Li Ka Shing Knowledge Institute, St Michael's Hospital, Unity Health Toronto, Canada

^*^share co-last authorship

**Corresponding author**

Emanuele Rezoagli, MD, PhD

School of Medicine and Surgery

University of Milano-Bicocca, 20900, Monza, Italy

Email: [emanuele.rezoagli@unimib.it](mailto:emanuele.rezoagli@unimib.it)

Phone: +390392339273

**Methods**

**Patients, study design and setting**

These are prespecified secondary analyses of an international, multicenter, randomized clinical trial (NCT03201263) (1) aimed at exploring physiological subgroups of patients potentially responsive to SIGH in terms of outcomes and whether differences in respiratory physiology might have a role as underpinning mechanisms of outcomes differences by using SIGH.

The PROTECTION trial included 20 centers from 8 countries between December 2017 to May 2019 through a call of the Pleural Pressure Working Group (PLUG) of the European Society of Intensive Care Medicine (ESICM) who endorsed and partially funded the trial.

The PROTECTION trial included patients with acute hypoxemic respiratory failure (AHRF) (PaO_2_/ FiO_2_ ≤ 300 with a PEEP of 5 cmH_2_O) who were mechanically ventilated between 24 h and 7 days and who were switched from mechanical ventilation to pressure support ventilation between 4-24 hours. Furthermore, at the enrolment, the Richmond Agitation-Sedation Scale was -2 to 0 (2).

The study was conducted according to the principles of the Declaration of Helsinki, and it was approved by the Ethics Committee of the Coordinating Center Fondazione IRCCS Ca’ Granda Ospedale Maggiore Policlinico of Milan, Italy (ref. 318/2017). The institutional review boards of all participating centers approved the trial. Informed consent was obtained for each patient according to local regulations. Further details about study design, population, exclusion criteria and methods were previously described (3).

**~~Randomization and interventions~~**

~~Patients were randomized to PSV with SIGH (SIGH group) or to PSV with no SIGH (no-SIGH group) after testing responsivity to SIGH test, a 30-min test where patients were exposed for 30 minutes to SIGH (i.e. 30 cmH~~_~~2~~_~~O for 3-s insufflation one each minute) starting with a FiO2 tailored to target a SpO2 between 90-96%. After the SIGH test, patients were defined as SIGH responders versus SIGH non-responders whether SpO2/FiO2 improved by >1%.~~

~~PSV setting after randomization targeted a Vt 6-8 mL/kg of predicted body weight, respiratory rate (RR) 20-35 breaths/minute, while clinical PEEP and FiO2 were unchanged. Subsequent changes in PSV, were considered at least every 8 h to reach the randomization target of Vt and RR, while PEEP and FiO2 were adjusted to maintain SpO2 90-96%.~~

~~A spontaneous breathing trial (SBT) was considered if SpO2 ≥90% on FiO2≤0.4 and PEEP≤5 cmH2O with no agitation and unstable hemodynamics.~~

~~Comprehensive information on randomization, interventions and SBT was previously described (3).~~

***Sigh test, randomization, ~~and~~ interventions and spontaneous breathing trial***

**All enrolled patients underwent a responsivity test to Sigh. Specifically, patients were exposed for 30 minutes to Sigh (i.e. 30 cmH_2_O for 3-s insufflation one each minute) starting with a FiO_2_ tailored to target a SpO_2_ between 90-96%. After the Sigh test, patients were defined as Sigh responders versus Sigh non-responders whether SpO_2_/FiO_2_ improved by >1%.**

After completion of the Sigh test, patients were randomized to PSV with Sigh (Sigh group) or to PSV with no sigh (No Sigh group).

~~after testing responsivity to Sigh test, a 30-min test where patients were exposed for 30 minutes to Sigh (i.e. 30 cmH~~_~~2~~_~~O for 3-s insufflation one each minute) starting with a FiO~~_~~2~~_ ~~tailored to target a SpO2 between 90-96%. After the SIGH test, patients were defined as Sigh responders versus Sigh non-responders whether SpO~~_~~2~~_~~/FiO~~_~~2~~_ ~~improved by >1%.~~

PSV setting after randomization targeted a Vt 6-8 mL/kg of predicted body weight, respiratory rate (RR) 20-35 breaths/minute, while clinical PEEP and FiO_2_ were unchanged.

**In the Sigh group, Sigh was promptly added as a pressure control breath at total end-inspiratory**

**pressure of 30 cmH_2_O for 3 s delivered once per minute. Ventilators were switched to biphasic synchronized positive airway pressure mode (also known as synchronized intermittent mandatory ventilation combining pressure control and PSV) with the lower pressure level set at clinical PEEP and the higher pressure level set at 30 cmH_2_O with a 3-s inspiratory time. Sigh settings were left unchanged until switch to controlled ventilation, day 28, death, or performance of a successful spontaneous breathing trial. In the No Sigh group, after randomization, PSV was set to obtain the same targets as above with clinical PEEP and the FiO_2_ selected during the prerandomization sigh test.** Subsequent changes in PSV **in both groups**, were considered at least every 8 h to reach the randomization target of Vt and RR, while PEEP and FiO_2_ were adjusted to maintain SpO_2_ 90-96%. **In both groups, switch to protective controlled ventilation was considered when in the presence of specific predefined criteria. Patients switched to controlled ventilation were reassessed at least every 8 h and switched back to the Sigh or No Sigh group as soon as predefined criteria for improvement were met. (3**)

**A spontaneous breathing trial (SBT) was considered if SpO_2_ ≥90% on FiO_2_≤0.4 and PEEP≤5 cmH_2_O with no agitation and unstable hemodynamics. In the sigh group, the attending physician withdrew sigh, waited 60 min, confirmed the above-mentioned criteria, and performed the SBT. If criteria were no longer met, sigh was reintroduced and this procedure was repeated after at least 8 h. The SBT lasted at least 60 min with a combination of PEEP of 0 to 5 cmH_2_O and PSV level of 0 to 5 cmH_2_O. Criteria for success vs failure of the SBT were predefined by study protocol. (3) After successful completion of the SBT, patients were promptly extubated or, in the presence of tracheostomy, mechanical ventilation was discontinued. After SBT failure, patients were switched back to the Sigh or No Sigh group, and criteria for SBT were checked again after at least 6 h. After extubation, reintubation was performed if at least one of the criteria predefined by the study protocol was present. (3)**

Comprehensive information on randomization, interventions and SBT was previously described (3).

**Study hypothesis and ~~study subgroups~~ predefined physiological subgroups**

These analyses may serve as exploratory and hypotheses generating to understand whether the use of sigh may be a ventilatory option based on the physiological response in oxygenation to sigh and in regard to the set levels of PEEP. We based our analyses on a physiological rationale.

We started from the hypothesis that sigh breathing may be beneficial on outcome in the presence of oxygenation response during the sigh test (responders) – which was defined by SpO_2_/FiO_2_ criteria >1% – as compared with No Sigh. **Therefore, predefined subgroups responders versus non-responders were defined based on the 30-minutes Sigh test according to oxygenation criteria. We explored differences on outcome between Sigh and No Sigh treatment In responders and non-responders subgroups.**

Subsequently, we hypothesized that patients exposed to low levels of PEEP (PEEP≤8 cmH_2_O - PEEP=8cmH2O first quartile in the PROTECTION trial - Low PEEP group) may show a lower mortality rate by adding Sigh as compared with No Sigh.

In the presence of mortality differences between Sigh and No Sigh, we explored daily differences in physiological parameters between the 2 randomized groups, and whether physiological parameters were associated with outcomes. **The specific cut-off used to define High versus Low PEEP group was decided based on statistical reasons (i.e. to obtain balanced samples between the 2 predefined subgroups) and on baseline oxygenation criteria (i.e. patients with mild hypoxemia, average 200<PaO_2_/FiO_2_≤300).**

**Measurements and study outcomes**

After enrolment and at randomization, data on demographics, past and recent medical history, systemic severity, lung injury risk factors, ventilation clinical settings and etiology of AHRF were collected. Furthermore, daily physiological measurements were collected during the first 7 days after randomization. Study outcomes including 28-day mortality and successful extubation with more than 48 hours free from reintubation at 28-day and data on sigh feasibility were explored.

**Statistical analysis**

Continuous data were described with median and quartiles (Q_1_–Q_3_). Categorical data were reported as count (proportion). Descriptive statistics were used to characterize the study population. A two-tailed p-value below 0.05 was considered statistically significant. Differences between the randomized groups (SIGH versus no-SIGH groups) are reported by Mann-Whitney Wilcoxon-test and by Chi-square or Fisher’s exact test, as appropriate.

Differences in 28-day mortality and successful extubation with more than 48 hours free from reintubation at 28-day were evaluated by survival curves using the Kaplan-Meier approach with log-rank p-value and competing risk non-parametric method with Fine & Gray p-value, respectively. The association of the study intervention (SIGH versus no-SIGH) with 28-day mortality and successful extubation was investigated by using multivariable Cox-proportional and Fine & Gray models using mortality as a competitive event, respectively. **The number of covariates used to adjust the multivariable model for the explored outcomes were decided based on the explored outcome of the sample size of to avoid overfitting. The specific covariates for multivariable adjustment were decided based on clinical meaning and their known association with outcomes including:** ~~Clinically meaningful variables used to adjust the multivariable models included:~~

- Age;
- **Patient** past medical history **– that was described** by ~~using~~ the presence of **any** comorbidities **among the following ones** ~~(i.e.~~ Chronic cardiovascular disease, Chronic pulmonary disease, Diabetes, Chronic renal disease, Cancer); and
- **Patients current** clinical illness severity by using SOFA score.

Results of the multivariate models were reported as β coefficient, Hazard Ratio (HR) with 95% CI (95% CI).

Daily differences up to 7 days since randomization in physiological variables between the study interventions (SIGH versus no-SIGH) in the investigated physiological subgroups were assessed by using generalized estimating equation models account for repeated measures for subjects. Association between average physiological parameters within 7-d and study outcomes were performed by using Cox-proportional (i.e. 28-day mortality) and Fine & Gray models using mortality as a competitive event (i.e. successful extubation with more than 48 hours free from reintubation at 28-day). Differences in ventilatory ratio between survivors and non-survivors were assessed by using Mann-Whitney U-test. Statistical analyses were performed with SAS 9.4 TS Levek 1M7 (2020 SAS Institute Inc., Cary, NC, USA) and R Studio 2002.07.1 (2009-2002Rstudio PBC).

**References**

1. Mauri T, Foti G, Fornari C, Grasselli G, Pinciroli R, Lovisari F, Tubiolo D, Volta CA, Spadaro S, Rona R, Rondelli E, Navalesi P, Garofalo E, Knafelj R, Gorjup V, Colombo R, Cortegiani A, Zhou JX, D'Andrea R, Calamai I, Vidal González Á, Roca O, Grieco DL, Jovaisa T, Bampalis D, Becher T, Battaglini D, Ge H, Luz M, Constantin JM, Ranieri M, Guerin C, Mancebo J, Pelosi P, Fumagalli R, Brochard L, Pesenti A; PROTECTION Trial Collaborators. Sigh in Patients With Acute Hypoxemic Respiratory Failure and ARDS: The PROTECTION Pilot Randomized Clinical Trial. Chest. 2021 Apr;159(4):1426-1436. doi: 10.1016/j.chest.2020.10.079.
2. Sessler CN, Gosnell MS, Grap MJ, Brophy GM, O'Neal PV, Keane KA, Tesoro EP, Elswick RK. The Richmond Agitation-Sedation Scale: validity and reliability in adult intensive care unit patients. Am J Respir Crit Care Med. 2002 Nov 15;166(10):1338-44. doi: 10.1164/rccm.2107138.
3. Mauri T, Foti G, Fornari C, Constantin JM, Guerin C, Pelosi P, Ranieri M, Conti S, Tubiolo D, Rondelli E, Lovisari F, Fossali T, Spadaro S, Grieco DL, Navalesi P, Calamai I, Becher T, Roca O, Wang YM, Knafelj R, Cortegiani A, Mancebo J, Brochard L, Pesenti A; Protection Study Group. Pressure support ventilation + sigh in acute hypoxemic respiratory failure patients: study protocol for a pilot randomized controlled trial, the PROTECTION trial. Trials. 2018 Aug 29;19(1):460. doi: 10.1186/s13063-018-2828-8.

**Supplemental Table 1. Outcomes and feasibility of non-responders randomized to Sigh.**

|  | **No Sigh**  **(N=46)** | **Sigh**  **(N=56)** | | **p-value** |
| --- | --- | --- | --- | --- |
| Deaths ICU – N (%) | 13 (28) | 8 (14) | | 0.0824 |
| Deaths at 28 days – N (%)° | 16 (36) | 9 (17) | | 0.0288 |
| Successful Extubation – N (%) | 30 (65) | 47 (84) | | 0.0288 |
| Non-extubated – survivors | 6 (13) | | 3 (5) | 0.0469 |
| Non-extubated – dead at 28 days | 10 (22) | | 6 (11) |  |
| Successfully Extubated – survivors | 24 (52) | | 44 (79) |  |
| Successful Extubated – dead at 28 days | 6 (13) | 3 (5) | |  |
| VFD - median (Q_1_ - Q_3_) | 10 (0-24) | 23 (8.5-26) | | 0.0074 |
| VFD in 28-day survivors - median (Q_1_ - Q_3_) | 22 (5-26) | 25 (19-26) | | 0.0805 |
| Days in CV - median (Q_1_ - Q_3_) | 0 (0-1) | 0 (0-0) | | 0.0522 |
| ICU Discharge, patients discharged alive from ICU - N (%) |  |  | |  |
| Home | 0 (0) | 3 (7) | | 0.4058 |
| Other ICU | 4 (14) | 1 (2) | |  |
| Other Ward | 24 (86) | 42 (92) | |  |
| Rehabilitation Facility | 0 (0) | 0 (0) | |  |
| Days in ICU^1^ - median (Q_1_ - Q_3_) | 8.5 (5-16) | 6 (3-12.5) | | 0.1502 |
| Tracheostomy - N (%) | 13 (28) | 14 (25) | | 0.7103 |
| Switch to other study ARM - N (%) | 2 (4) | 2 (4) | | 1.000 |
| **Feasibility** |  |  | |  |
| Switch to CV ≥ 24 h | 11 (24) | 6 (10) | | 0.0751 |
| Rescue treatment | 9 (20) | 4 (7) | | 0.0612 |
| Reintubation within 48 hours^2^ | 6 (13) | 5 (9) | | 0.5377 |
| At least one of above events^2^ | 16 (35) | 13 (23) | | 0.1975 |
| Reason of first Switch CV – N (%) |  |  | |  |
| PSV support>20 cmH_2_O AND/OR Ph<7.3 | 5 (11) | 0 (0) | | 0.0511 |
| PEEP≥15 cmH20 and/or PaO2/Fio2≤100 mmHg | 4 (9) | 4 (7) | |  |
| Uncontrolled Hypotension/hypertension | 0 (0) | 0 (0) | |  |
| Active cardiac ischemia/unstable arrhythmias | 1 (2) | 0 (0) | |  |
| Abrupt decrease of consciusness level (RASS<-3)  or dangerous agitation (RASS>2) | 0 (0) | 2 (4) | |  |
| Necessity to perform diagnostic test | 1(2) | 0 (0) | |  |
| Type of first Rescue treatment (new Classification) – N (%) |  |  | |  |
| Recruitment manouvers | 5(11) | 3 (5) | | 0.2297 |
| PEEP≥15 cm H_2_O | 2 (4) | 1 (2) | |  |
| Prone position | 2 (4) | 0 (0) | |  |
| Adverse events – N (%) | 9 (20) | 7 (13) | | 0.3289 |
| Type of first Adverse Event (New Classification) – N (%) |  |  | |  |
| Hemodynamic instability | 5 (11) | 3 (5) | | 0.6571 |
| Arrhythmias | 3 (7) | 2 (4) | |  |
| Barotrauma | 1 (2) | 2 (4) | |  |

^1^From Randomization; ^2^Excluding events with tracheostomy.

CV=controlled ventilation; ICU=intensive care unit; PaO_2_=arterial oxygen partial pressure; PEEP=positive end-expiratory pressure; PSV=pressure support ventilation; RASS=Richmond Agitation-Sedation Scale; VFD=ventilator free days.

**Supplemental Table 2. Outcomes of pts with low PEEP randomized to Sigh.**

|  | **No Sigh**  **(N=59)** | **Sigh (N=54)** | | **p-value** |
| --- | --- | --- | --- | --- |
| Deaths ICU – N (%) | 13 (22) | 5 (9) | | 0.0638 |
| Deaths at 28 days – N (%)° | 18 (31) | 7 (13) | | 0.0248 |
| Successful Extubation – N (%) | 42 (72) | 47 (87) | | 0.1409 |
| Non-extubated – survivors | 6 (10) | | 2 (4) | 0.0460 |
| Non-extubated – dead at 28 days | 11 (19) | | 5 (9) |  |
| Successfully Extubated – survivors | 35 (59) | | 45 (83) |  |
| Successfully Extubated – dead at 28 days | 7 (12) | 2 (4) | |  |
| VFD - median (Q_1_ - Q_3_) | 22 (0-26) | 25 (9-27) | | 0.1000 |
| VFD in 28-day survivors - median (Q_1_ - Q_3_) | 25 (21-26) | 26 (19-27) | | 0.5955 |
| Days in CV - median (Q_1_ - Q_3_) | 0 (0-0) | 0 (0-0) | | 0.9287 |
| ICU Discharge, patients discharged alive from ICU - N (%) |  |  | |  |
| Home | 2 (5) | 3 (6) | | 0.1702 |
| Other ICU | 6 (14) | 1 (2) | |  |
| Other Ward | 35 (80) | 42 (89) | |  |
| Rehabilitation Facility | 1 (3) | 1 (2) | |  |
| Days in ICU^1^ - median (Q_1_ - Q_3_) | 6 (4-9) | 5 (3-14) | | 0.8493 |
| Tracheostomy - N (%) | 8 (14) | 12 (22) | | 0.2281 |
| Switch to other study ARM - N (%) | 1 (2) | 2 (4) | | 0.6054 |
| **Feasibility** |  |  | |  |
| Switch to CV ≥ 24 h | 10 (17) | 6 (11) | | 0.3739 |
| Rescue treatment | 6 (10) | 2 (4) | | 0.2751 |
| Reintubation within 48 hours^2^ | 5 (9) | 6 (11) | | 0.6367 |
| At least one of above events^2^ | 15 (25) | 10 (19) | | 0.3771 |
| Reason of first Switch CV – N (%) |  |  | |  |
| PSV support>20 cmH_2_O AND/OR Ph<7.3 | 4 (7) | 2 (4) | | 0.9230 |
| PEEP≥15 cmH20 and/or PaO2/Fio2≤100 mmHg | 2 (4) | 2 (4) | |  |
| Uncontrolled Hypotension/hypertension | 0 (0) | 0 (0) | |  |
| Active cardiac ischemia/unstable arrhythmias | 0 (0) | 0 (0) | |  |
| Abrupt decrease of consciousness level (RASS<-3)  or dangerous agitation (RASS>2) | 3 (6) | 2 (4) | |  |
| Necessity to perform diagnostic test | 1 (2) | 0 (0) | |  |
| Type of first Rescue treatment (new Classification) – N (%) |  |  | |  |
| Recruitment manouvers | 3 (5) | 2 (4) | | 0.6808 |
| PEEP≥15 cm H_2_O | 1 (2) | 0 (0) | |  |
| Prone position | 2 (4) | 0 (0) | |  |
| Adverse events – N (%) | 9 (20) | 7 (13) | | 0.3289 |
| Type of first Adverse Event (New Classification) – N (%) |  |  | |  |
| Hemodynamic instability | 5 (9) | 2 (4) | | 0.5583 |
| Arrhythmias | 3 (5) | 1 (2) | |  |
| Barotrauma | 1 (2) | 1 (2) | |  |

^1^From Randomization; ^2^Excluding events with tracheostomy.

CV=controlled ventilation; ICU=intensive care unit; PaO_2_=arterial oxygen partial pressure; PEEP=positive end-expiratory pressure; PSV=pressure support ventilation; RASS=Richmond Agitation-Sedation Scale; VFD=ventilator free days.

**Supplemental Table 3. Exploratory differences in the proportion of 28-day mortality and successful extubation by competing risk analyses between predefined physiological subgroups exposed or not exposed to Sigh.**

| **A) 28-day mortality**  **Log-rank p-value** | **Non-responders-No Sigh** | **Non-responders- Sigh** | **Responders-**  **No Sigh** | **Responders-**  **Sigh** |
| --- | --- | --- | --- | --- |
| **Non-responders-**  **No Sigh** |  | 0.0311 | 0.0042 | 0.0222 |
| **Non-responders-**  **Sigh** |  |  | 0.6489 | 0.9694 |
| **Responders-**  **No Sigh** |  |  |  | 0.5879 |
| **Responders-**  **Sigh** |  |  |  |  |

| **B) 28-day mortality**  **Log-rank p-value** | **Low PEEP-No Sigh** | **Low PEEP- Sigh** | **High PEEP-No Sigh** | **High PEEP - Sigh** |
| --- | --- | --- | --- | --- |
| **Low PEEP-No Sigh** |  | 0.0209 | 0.0142 | 0.1314 |
| **Low PEEP-Sigh** |  |  | 0.9731 | 0.334 |
| **High PEEP-No Sigh** |  |  |  | 0.3248 |
| **High PEEP-Sigh** |  |  |  |  |

| **C) 28-day successful extubation**  **Fine&Gray p-value** | **Non-responders-No Sigh** | **Non-responders- Sigh** | **Responders-No Sigh** | **Responders- Sigh** |
| --- | --- | --- | --- | --- |
| **Non-responders-**  **No Sigh** |  | 0.0238 | 0.0129 | 0.049 |
| **Non-responders- Sigh** |  |  | 0.9621 | 0.6479 |
| **Responders-**  **No Sigh** |  |  |  | 0.6591 |
| **Responders- Sigh** |  |  |  |  |

| **D) 28-day successful extubation**  **Fine&Gray p-value** | **Low PEEP-No Sigh** | **Low PEEP-Sigh** | **High PEEP-No Sigh** | **High PEEP-Sigh** |
| --- | --- | --- | --- | --- |
| **Low PEEP-No Sigh** |  | 0.0612 | 0.7043 | 0.839 |
| **Low PEEP-Sigh** |  |  | 0.1088 | 0.0738 |
| **High PEEP-No Sigh** |  |  |  | 0.8367 |
| **High PEEP-Sigh** |  |  |  |  |

**
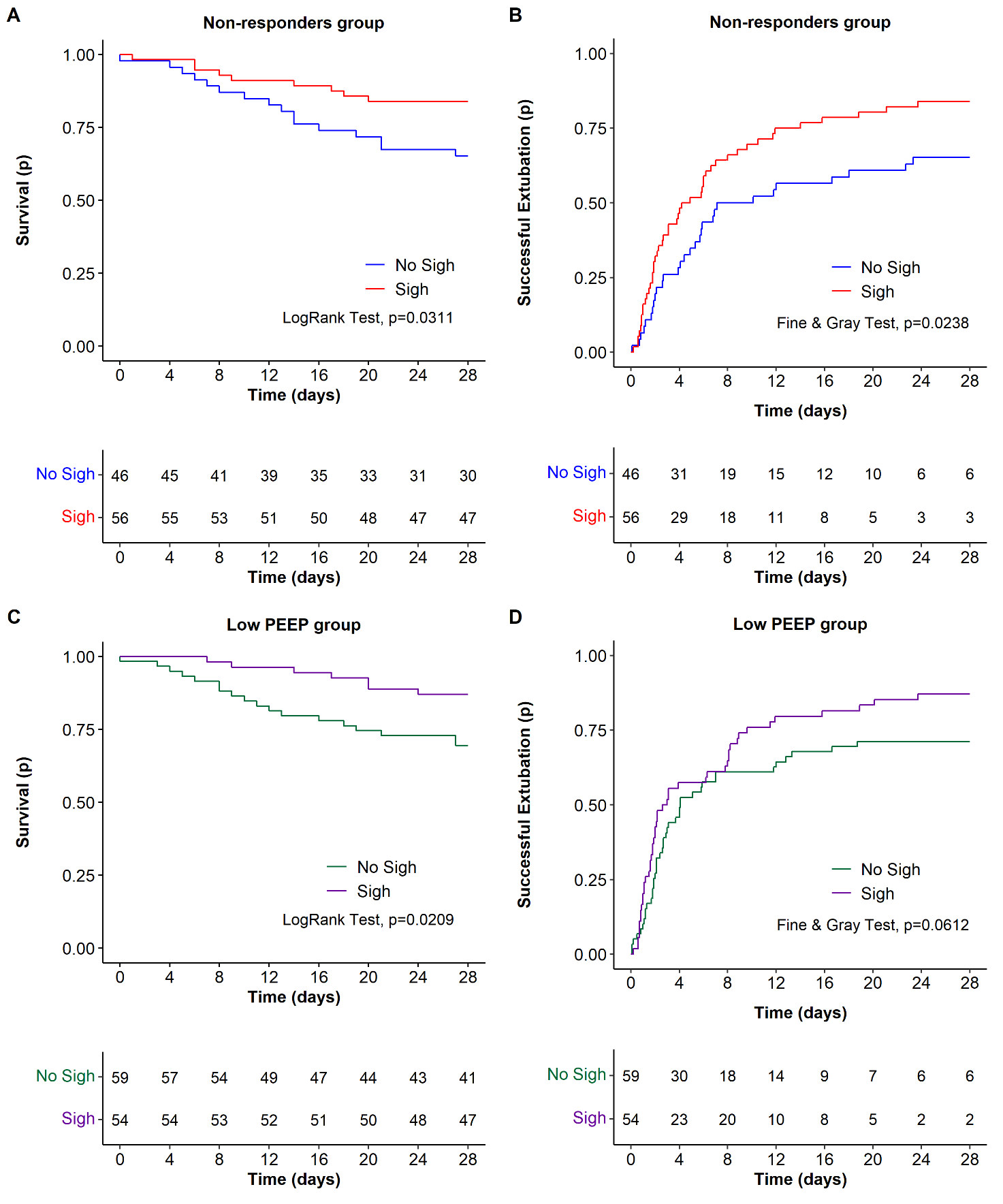
**

**Supplemental Figure 1.** Death at 28-day follow-up (A) and time to successfull extubation (B) stratified by SIigh versus no SIGH arm in the Non-responders group. Death at 28-day follow-up (C) and time to successful extubation≥48 hours (D) stratified by Sigh versus no Sigh group in the Low PEEP group.

**
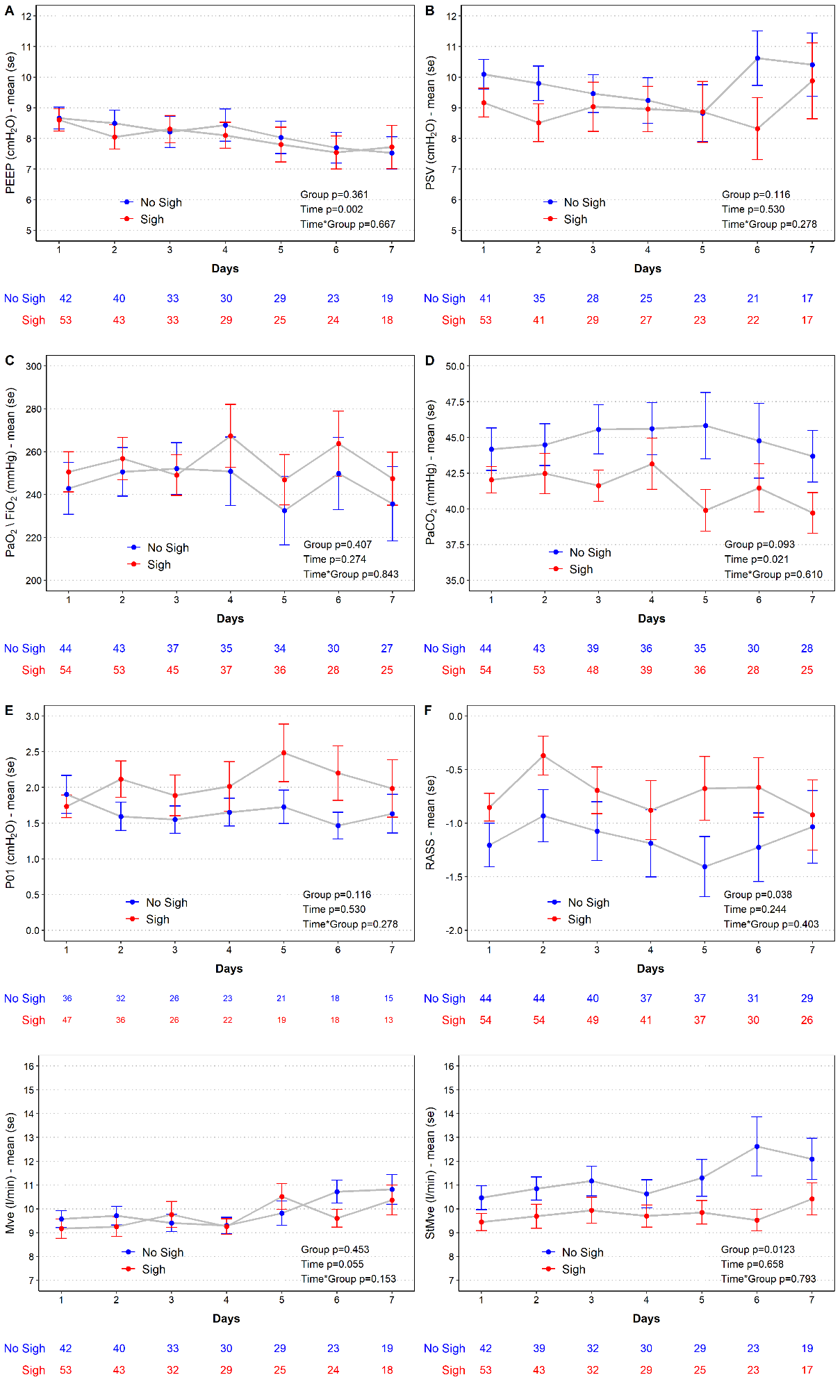
**

**Supplemental Figure 2.** PEEP (A), Pressure support level (B), PaO2/FiO2 (C), PaCO2 (D), p0.1 (E), RASS (F), minute ventilation (G) and standardized minute ventilation (H) differences over 7-day follow up between Sigh versus No Sigh arm in the non-responders group.

**
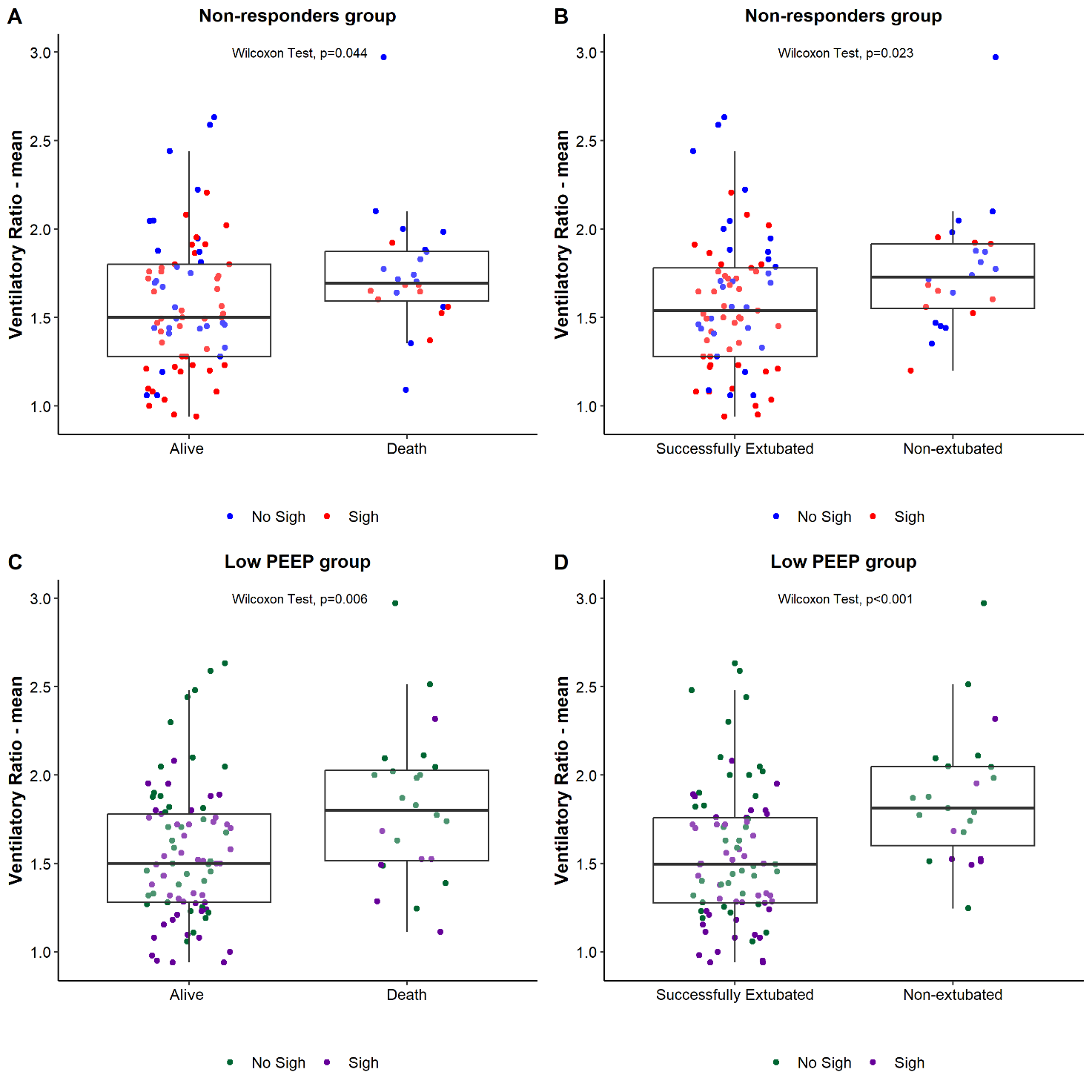
**

**Supplemental Figure 3.** Differences in Ventilatory Ratio between survivors and non-survivors (A) and between successfully extubated and non-extubated (B) in the Non-responders group. Differences in Ventilatory Ratio between survivors and non-survivors (C) and between successfully extubated and non-extubated s (D) in the Low PEEP group.

**
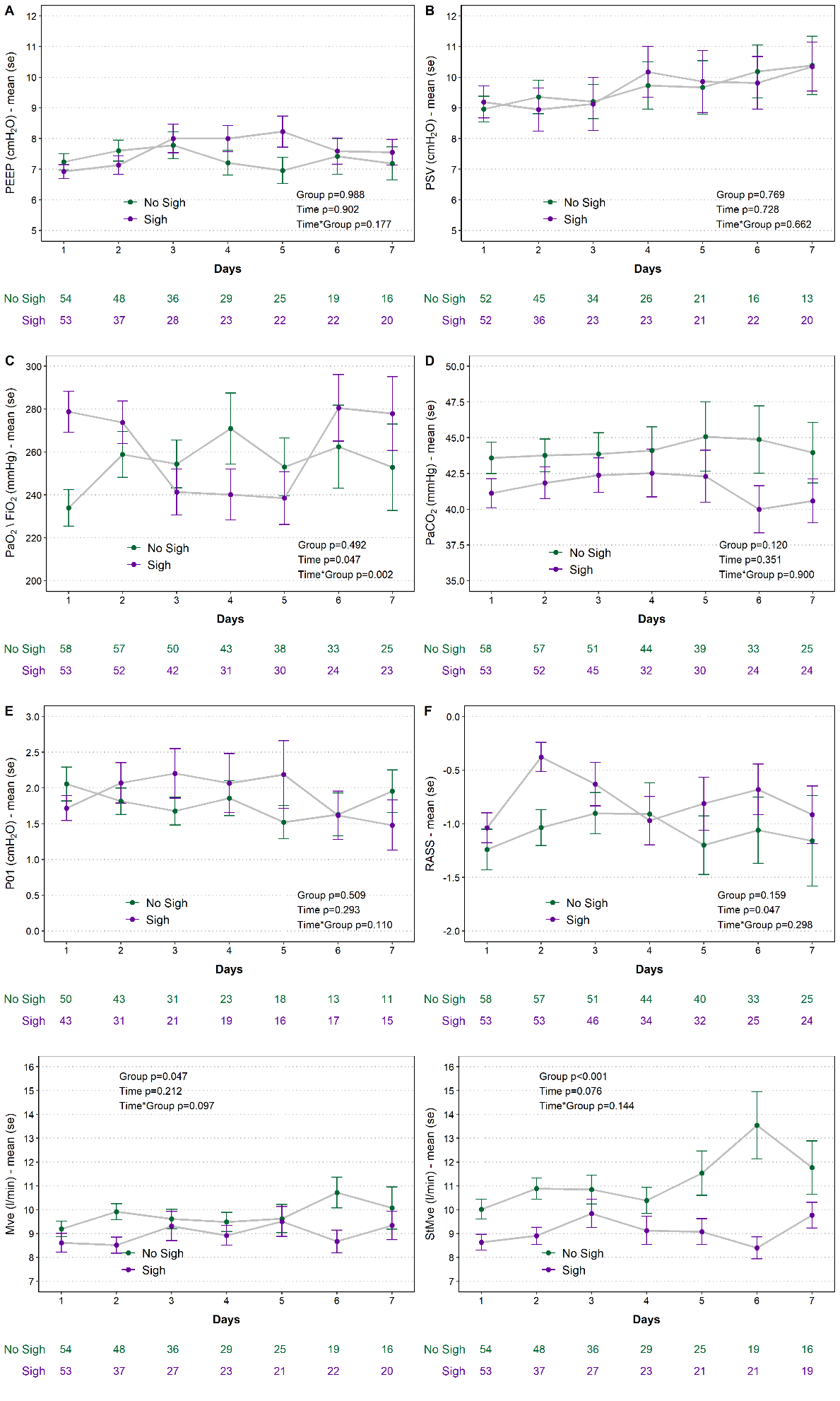
**

**Supplemental Figure 4.** PEEP (A), Pressure support level (B), PaO2/FiO2 (C), PaCO2 (D), p0.1 (E), RASS (F), minute ventilation (G) and standardized minute ventilation (H) differences over 7-day follow up between Sigh versus No Sigh arm in the Low PEEP group.
